# The Association between Environmental Lead Exposure and Bone Density in Children

**DOI:** 10.1289/ehp.6555

**Published:** 2004-04-07

**Authors:** James R. Campbell, Randy N. Rosier, Leonore Novotny, J. Edward Puzas

**Affiliations:** ^1^Department of Pediatrics and; ^2^Department of Orthopedics, University of Rochester Medical Center, Rochester, New York, USA

**Keywords:** blood lead levels, bone mineral density, dual-energy X-ray absorptiometry, parathyroid hormone–related peptide

## Abstract

Osteoporosis is a decrease in bone mineral density (BMD) that predisposes individuals to fractures. Although an elderly affliction, a predisposition may develop during adolescence if a sufficient peak BMD is not achieved. Rat studies have found that lead exposure is associated with decreased BMD. However, human studies are limited. We hypothesized that the BMD of children with high lead exposure would be lower than the BMD of children with low lead exposure. We collected data on 35 subjects; 16 had low cumulative lead exposure (mean, 6.5 μg/dL), and 19 had high exposure (mean, 23.6 μg/dL). All were African American; there was no difference between the groups by sex, age, body mass index, socioeconomic status, physical activity, or calcium intake. Significant differences in BMD between low and high cumulative lead exposure were noted in the head (1.589 vs. 1.721 g/cm^2^), third lumbar vertebra (0.761 vs. 0.819 g/cm^2^), and fourth lumbar vertebra (0.712 vs. 0.789 g/cm^2^). Contrary to our hypothesis, subjects with high lead exposure had a significantly higher BMD than did subjects with low lead exposure. This may reflect a true phenomenon because lead exposure has been reported to accelerate bony maturation by inhibiting the effects of parathyroid hormone–related peptide. Accelerated maturation of bone may ultimately result in a lower peak BMD being achieved in young adulthood, thus predisposing to osteoporosis in later life. Future studies need to investigate this proposed model.

Research on the adverse effects of lead exposure on humans has focused on neurocognitive outcomes among children [Canfield et al. 2003; National Research Council (NRC) 1993]. However, a growing body of literature reports that the effects of childhood lead exposure continue into adolescence and adulthood. These include delinquent behavior (Dietrich et al. 2001; Needleman et al. 2002), dental caries (Moss et al. 1999), hypertension (Hu et al. 1996; Nash et al. 2003), cardiac arrhythmias (Cheng et al. 1998), and renal dysfunction (Kim et al. 1996). Research also demonstrates another potential late effect of childhood lead exposure: osteoporosis. Studies on rats have found that increased lead exposure is associated with decreased bone density (Escribano et al. 1997; Gruber et al. 1997; Puzas et al. 1999) and decreased bone strength (Ronis et al. 2001). Additional studies have found that lead exposure inhibits the function of osteoblasts (Klein and Wiren 1993; Puzas et al. 1999; Ronis et al. 2001), the cells that make bone.

However, human studies on this association are limited. In a study of children, Laraque et al. (1990) found no association between bone density and lead exposure. However, because the comparison group was made up of children with moderate-level lead exposure (i.e., blood lead level 12–29 μg/dL), such a study cannot exclude the possibility that lead exposure has an effect at lower levels. In addition, because the children were examined at a young age (range, 18–47 months), sufficient time may not have elapsed for the adverse effects on the bone to become manifest. A study by Alfvén et al. (2002) also found no association between lead exposure and bone mineral density (BMD) in a cross-sectional study of adults. However, the authors used concurrent blood lead level to define lead exposure and acknowledged that such a measure may be inadequate to measure body lead burden. In addition, subjects did not have high blood lead levels [the mean blood lead level was 3.1 μg/dL (Alfvén T, personal communication)]; thus, the lack of an association may be due to low lead exposure among the subjects.

Our objective was to determine whether an association between lead exposure and bone density exists in children. We hypothesized that the bone density of children with high lead exposure would be lower than the bone density of children with low lead exposure.

## Materials and Methods

### Subject identification and enrollment.

To identify potential subjects who had an adequate number of blood lead levels to define past lead exposure, we obtained a comprehensive database of blood lead levels from the local county health department (Department of Health, Monroe County, New York State). To minimize the effect of age on BMD, we limited the database to children 8–10 years of age. In the database, we excluded capillary blood lead levels ≥ 10 μg/dL, because of the possibility that these represent contaminated specimens [Centers for Disease Control and Prevention (CDC) 1991], and children who did not have at least one blood lead level at each of four age groups (13–24 months, 25–36 months, 37–48 months, and 49–60 months). For the remaining children, we calculated each child’s cumulative lead exposure (defined below) and created a list with the following information: child’s name, date of birth, race, and cumulative lead exposure. From the list, we identified children who attended the principal investigator’s (J.R.C.) pediatric practice or whom the principal investigator had medically managed for lead toxicity. To eliminate the effect of race, we contacted only subjects who were African American. The principal investigator (J.R.C.) subsequently called the parents of the potential subjects to ask if they would be interested in having the child enrolled. If a parent agreed, a short questionnaire was administered to determine whether exclusionary criteria existed. We excluded children who had medical conditions that affected bone density (metabolic bone disease, renal disease, sickle cell disease), used certain medications (corticosteroids, anticonvulsants, diuretics), had evidence of sexual maturation (i.e., Tanner stage ≥ 2), or had a parent who was not African American. If no exclusionary criteria existed, the study coordinator (L.N.) called the parent to schedule an appointment for the bone density procedure. At the appointment, the study coordinator obtained informed consent, completed a questionnaire to collect covariate date, and measured the child’s height and weight. Subsequently, one technician conducted the bone density measurement. The study coordinator and technician were blinded to the subject’s cumulative lead exposure status.

The Human Subjects Committee of the Monroe County Health Department, the Human Subjects Review Board of the University of Rochester Medical Center, and the Clinical Investigation Committee of Rochester General Hospital approved this study.

### Measure of lead exposure.

The measure of lead exposure used in this study is termed the cumulative lead exposure. To compute it, we identified all blood lead levels collected during four age strata (i.e., 12–23 months, 24–35 months, 36–47 months, and 48–60 months) from the local health department database. Subsequently, we calculated the arithmetic mean of all blood lead levels for each of the four age strata. Finally, the cumulative lead exposure was calculated by computing the arithmetic mean of the four age strata means. Subjects were dichotomized as having high versus low cumulative lead exposure at a cutoff of 15 μg/dL.

There is a strong correlation (i.e., 0.92–0.95) between any single blood lead level in children between 2 and 4 years of age and cumulative lead exposure measure based on 24 serial blood lead levels in children between 3 months and 10 years of age (Dietrich K, personal communication); therefore, we conclude that our measure of cumulative lead exposure is a valid measure of the overall lifetime lead exposure for a school-age child.

### Measure of bone density.

We used a fan-beam dual-energy X-ray absorptiometry (DEXA) scanner (Prodigy; GE/Lunar Corporation, Madison, WI) to measure BMD (Mazess et al. 1990). BMD was determined at various body regions (e.g., total body, arms, legs, trunk), the lumbar vertebrae, and hip regions (total hip, femoral neck, trochanter, femoral shaft).

### Measure of covariates.

Variables associated with changes in bone density include age (Boot et al. 1997; Maynard et al. 1998), race (Nelson et al. 1997; Pollitzer and Anderson 1989), weight (Boot et al. 1997), physical activity (Cooper et al. 1988), and calcium intake (Johnston et al. 1992). Bone density does not vary by sex among children < 13 years of age (Maynard et al. 1998).

To minimize the effect of age, we enrolled only subjects within a narrow age range: 8–10 years. To eliminate the effect of race, we enrolled only subjects who were African American (and whose parents were both African American). We measured subject weight and height at the time of the BMD measurement. A parental questionnaire collected data on physical activity (i.e., the number of hours a day the child is physically active and inactive), calcium intake (i.e., current milk and milk-product intake and frequency), and socioeconomic status [head of household Hollingshead occupational level and socioeconomic score (Hollingshead 1958)].

### Analyses.

We initially compared the covariates between subjects by cumulative lead exposure status (i.e., low vs. high). Because age and weight are strongly associated with BMD, we decided, *a priori*, to introduce both into adjusted analyses. Other comparisons with *p*-values ≤0.20 were also to be introduced into adjusted analyses. The primary analysis was, for each bony site, a comparison of the mean BMD by cumulative lead exposure status. Using SPSS software (version 4.0; SPSS Inc., Chicago, IL), we conducted adjusted analyses by use of analysis of covariance between the cumulative lead exposure groups.

A sample size calculation demonstrated that 44 subjects would be required to achieve a power of 80% in discerning a 1.0-SD difference in BMD between the groups. We conducted analyses during subject recruitment, thus allowing us to discontinue enrollment after significant findings were discerned at a sample size of 35 subjects.

## Results

We collected data on 36 subjects. All were African American. One subject was excluded because of obesity [body mass index (BMI) = 33]. Among the remaining subjects, 63% were male. The mean age was 109.5 months. The mean weight was 33.6 kg, and the mean height 138.6 cm; these measures are approximately at the 75th and 60th percentiles, respectively.

Among the 35 subjects analyzed, 16 had a low cumulative lead exposure and 19 had a high cumulative lead exposure; the respective mean cumulative lead exposures were 6.5 μg/dL (range, 2.7–10.3 μg/dL) and 23.6 μg/dL (range, 15.5–43.5 μg/dL) (Table 1). The groups were otherwise comparable; there were no differences (i.e., *p* > 0.20) between the groups on sex distribution, age, BMI, socioeconomic status, physical activity, or calcium intake (Table 2).

Table 3 shows the adjusted mean BMD by bony site and cumulative lead exposure status. Contrary to our hypothesis, we found that subjects with high cumulative lead exposure had a higher BMD than did subjects with low cumulative lead exposure. Among 17 bony sites, four were significantly different (i.e., *p* ≤0.05).

## Discussion

Contrary to our hypothesis, we found that subjects with high lead exposure had a significantly higher bone density than did subjects with low lead exposure. We initially considered whether this result derived from an artificial increase in the measure of bone density by DEXA due to the presence of lead in bone. A false elevation of DEXA-based bone density is reported to occur in bone containing strontium, a heavy metal with a lower atomic weight than lead (Nielsen et al. 1999). We found, in an *in vitro* study using an older Lunar DPX-L pencil-beam instrument, that bone density increased by 8–11% with increasing and clinically relevant bone lead levels (i.e., 10–100 μg/g) (Puzas et al. 2002). However, when this *in vitro* study was replicated using a newer Lunar Prodigy fan-beam instrument, the same used on the subjects of our study, the effect was minimal and within the precision of the DEXA measure (Muzytchuk et al. 2004).

The alternative interpretation of our findings is that high lead exposure is associated with truly higher bone density in childhood. Our results indicate that the magnitude of this association is clinically relevant. For example, the mean BMD of the lumbar vertebrae (L1–L4) was 0.770 g/cm^2^ versus 0.720 g/cm^2^ among subjects with high and low cumulative lead exposure, respectively (*p* = 0.03). Thus, in this study, children with high cumulative lead exposure had nearly 7% higher BMD at the lumbar vertebrae than did children with low cumulative lead exposure. This amounts to about 2 years of bone growth.

We now wish to speculate on the mechanism of this finding. An *in vitro* study found that lead inhibits parathyroid hormone–related peptide (PTHrP) and transforming growth factor-β1, proteins that decrease the rate of maturation of chondrocytes in endochondral bone formation (Zuscik et al. 2002). Further, this inhibition of PTHrP is associated with accelerated skeletal maturity. Mice homozygous for PTHrP gene deletion have advanced skeletal maturation at birth (Karaplis et al. 1994; Lee et al. 1996). Similarly, children with Blomstrand syndrome, a congenital chondrodysplasia due to nonfunctioning PTHrP receptors (Jobert et al. 1998; Karaplis et al. 1998), also have advanced skeletal maturation and higher than normal bone density at birth (Blomstrand et al. 1985; den Hollander et al. 1997; Loshkajian et al. 1997; Young et al. 1993). The inhibition of PTHrP causes premature maturation of the chondrocytes (Zuscik et al. 2002), which may result in a higher bone density.

Our findings differ from past research findings that lead exposure is associated with lower, not higher, bone density in mature animals (Escribano et al. 1997; Gruber et al. 1997; Puzas et al. 1999; Ronis et al. 2001). Nevertheless, the literature suggests that the higher bone density associated with PTHrP inhibition is transient. Although mice homozygous for PTHrP gene deletion have higher bone density at birth (Karaplis et al. 1994), mice heterozygous for PTHrP gene deletion have osteopenia as adults (Amizuka et al. 1996; He et al. 2000). The proposed mechanism is as follows: Besides its effects on endochondral bone formation, PTHrP also acts on bone remodeling in adult organisms. It promotes the differentiation of bone marrow stem cells toward osteoblasts and away from adipocytes and impedes the apoptosis of osteoblasts (Karaplis 2001). In a mature organism without endochondral bone formation, PTHrP inhibition on bone remodeling would predominate—that is, differentiation of stem cells toward adipocytes and an increased rate of osteoblast apoptosis—thus predisposing to osteoporosis (Karaplis et al. 2001). We therefore speculate that a lead-exposed individual may undergo a higher rate of bone loss when older than would individuals without lead exposure.

**Figure 1:**
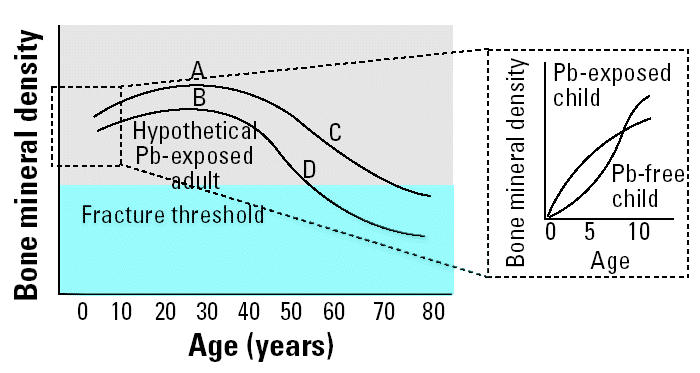
This study found that bone density for lead-exposed children is higher than that for children not exposed to lead. We propose that this increase may be transient (inset). A lower peak bone mass may occur in early adulthood (B rather than A), thus predisposing to osteoporosis in later life (D rather than C).

An alternative model for the development of osteoporosis is that a lead-exposed individual may achieve a lower peak bone mass as a young adult. Studies of children have found a negative association between blood lead level and height (Ballew et al. 1999; Schwartz et al. 1986; Selevan et al. 2003). Similarly, mice homozygous for PTHrP gene deletion, in addition to having advanced skeletal maturation, have shorter long bones and shorter vertebrae than do normal mice (Karaplis et al. 1994). These findings along with the findings described in the preceding paragraph suggest that lead targets its effects on the growth plate by inhibiting PTHrP and thus causing shorter stature in exposed children. We speculate that this inhibition of stature when young results in a lower peak bone mass being achieved, thus predisposing to osteoporosis in later life (Figure 1). Future studies are needed to investigate whether these proposed models are valid.

## Figures and Tables

**Table 1:** Blood lead level measures by cumulative lead exposure status [low vs. high (μg/dL)].

BLL measure	Low	High
Mean BLL		
12–23 months	7.3	23.8
24–35 months	7.4	22.4
36–47 months	5.4	24.5
48–60 months	4.9	21.1
Mean cumulative lead exposure*^a^*	6.5	23.6
Range	2.7–10.3	15.5–43.5

BLL, blood lead level.

a Defined in ”Materials and Methods.”

**Table 2:** Comparison of covariates by cumulative lead exposure status (low vs. high).

Covariates	Low	High	*p*-Value*^a^*
Demographics			
Sex (% male)	56	68	0.46
Age (months)	109.9	109.2	0.73
HOH Hollingshead occupation level*^b^*	5.6	6.3	0.30*^c^*
HOH Hollingshead socioeconomic score*^b^*	89.4	89.7	0.92
Body size			
Weight (kg)	34.1	33.2	0.72
Height (cm)	137.4	139.6	0.40
BMI (kg/m^2^)	17.9	16.9	0.28
Physical activity			
Active play (hr/day)	4.8	4.6	0.80
Inactive play (hr/day)	3.2	3.0	0.72
Calcium intake (portions/day)	3.6	3.6	1.00

HOH, head of household.

a By *t*-test, except where specified.

b HOH Hollingshead occupation level and socioeconomic level (Hollingshead 1958).

c By Mann-Whitney *U*-statistic.

**Table 3:** Adjusted BMD (g/cm^2^) by bony site and cumulative lead exposure (low vs. high).

BMD site	Low	High	*p*-Value*^a^*
Body regions			
Head	1.589	1.721	< 0.01**
Arms	0.684	0.704	0.16
Legs	0.917	0.928	0.61
Trunk	0.693	0.720	0.06*
Ribs	0.594	0.615	0.09*
Pelvis	0.806	0.839	0.09*
Spine	0.720	0.749	0.14
Total body	0.911	0.940	0.06*
Lumbar vertebrae			
L1*^b^*	0.682	0.707	0.28
L2	0.722	0.756	0.22
L3	0.761	0.819	0.01**
L4	0.712	0.789	0.01**
L1–L4	0.720	0.770	0.03**
Hip regions			
Femoral neck	0.827	0.893	0.07*
Trochanter	0.682	0.732	0.11
Femoral shaft	0.939	1.006	0.11
Total hip	0.842	0.906	0.08*

a By analysis of covariance.

b First lumbar vertebra.

* Marginally significant (i.e., 0.05 < *p* < 0.10).

** Significant at *p* ≤ 0.05 level.
